# Atheroprotective effects of pure tocotrienol supplementation in the treatment of rabbits with experimentally induced early and established atherosclerosis

**DOI:** 10.3402/fnr.v60.31525

**Published:** 2016-10-28

**Authors:** Thuhairah Abdul Rahman, Noor Faezah Hassim, Nurmazni Zulkafli, Suhaila Muid, Noor Kaslina Kornain, Hapizah Nawawi

**Affiliations:** 1 Faculty of Medicine, Universiti Teknologi MARA, Selangor, Malaysia; 2 Institute of Pathology, Laboratory and Forensic Medicine (I–PPerForM), Universiti Teknologi MARA, Selangor, Malaysia

**Keywords:** delta tocotrienol, gamma tocotrienol, atherosclerosis, hypercholesteroleamic rabbits, tissue biomarker

## Abstract

**Background:**

Atherosclerosis is the main cause of coronary artery disease -related deaths worldwide. The atheroprotective properties of pure tocotrienols (T3) in the absence of alpha-tocopherol (α-TCP) in vitamin E has not been extensively examined.

**Aim:**

To determine the atheroprotective properties of T3 in early and established atherosclerosis rabbits.

**Methods:**

Thirty New Zealand white rabbits were divided into two groups, B1 and B2 which represent early [fed 1% high cholesterol diet (HCD) for 2 weeks] and established (fed 1% HCD for 8 weeks) atherosclerosis. Each group was subdivided into three intervention arms: 1) T3–4 mg/kg, 2) T3–15 mg/kg and 3) vehicle without T3 (T3 negative) for 8 weeks. Serial fasting blood samples were obtained for lipid profile, and whole lengths of aorta were used to determine tissue markers of endothelial activation, inflammation and plaque stability.

**Results:**

In B1, atherosclerotic lesion in T3–4 mg/kg group was significantly reduced (*p*=0.008), while aortic tissue expression of vascular cellular adhesion molecule 1 (VCAM-1), interleukin-6 (IL-6) and matrix metalloproteinase (MMP-12) was reduced in T3–4 mg/kg compared to T3-negative rabbits group (0.2±0.1 vs. 28.5±3.1%; 3.0±1.6 vs. 14.0±1.7%; and 5.2±2.2 vs. 27.7±0.8%, respectively, *p<*0.05). T3–15 mg/kg group showed reduction in VCAM-1, E-selectin, IL-6 and MMP-12 (3.9±1.9 vs. 28.5±3.1%; 10.3±0.5 vs. 59.8±8.5%; 2.6±1.7 vs. 14.0±1.7%; and 16.2±3.2 vs. 27.7 0.8%, respectively, *p<*0.05). In B2, T3–4 mg/kg group reduced aortic tissue expression of intercellular adhesion molecule 1 (ICAM-1), E-selectin, IL-6, MMP-12 and MMP-9 compared to T3-negative rabbits group (29.9±2.4 vs. 55.3±1.3%; 26.7±1.5 vs. 60.5±7.6%; 15.7±0.7 vs. 27.7±4.8%; 34.8±2.7 vs. 46.5±3.4%; and 25.89±3.9 vs. 45.9±1.7%, respectively, *p<*0.05). T3–15 mg/kg group showed reduced VCAM-1, ICAM-1, E-selectin, IL-6, MMP-12 and MMP-9 (20.5±3.3 vs. 35.6±2.5%; 24.9±1.3 vs. 55.3±1.3%; 29.9±6.7 vs. 60.5±7.6; 11.3±2.2 vs. 27.7±4.8%; 23.0±1.7 vs. 46.5±3.4%; and 17.6±1.9 vs. 45.9±1.7%, respectively, *p<*0.05.

**Conclusion:**

These findings suggest the possible atheroprotective role T3 plays as an adjunct supplementation to standard treatment in the prevention of CAD.

Coronary artery disease (CAD) continues to remain the major cause of morbidity and mortality worldwide with 70% of cardiovascular disease (CVD)-related deaths being linked to atherosclerosis. WHO reported 30% of 71% total deaths due to chronic diseases in Malaysia in 2002 was mainly caused by CVD, and in 2008, 32% of 67% total non-communicable related deaths were due to CVD (1). Atherosclerosis is the main causative factor for CAD, which is known to be a chronic inflammatory disorder of the arterial wall characterized by progressive accumulation of lipids, inflammatory cells (macrophages, T-lymphocytes), smooth muscle cells and extracellular matrix known as atheromas or atherosclerotic plaques (2). There are several factors contributing to this disease including hypercholesterolaemia, hypertension, diabetes mellitus and smoking. Inflammatory response plays an important role in the onset, development, and evolution of atherosclerotic lesions (3). Controlling serum cholesterol levels through lipid-lowering medications such as statins has been the mainstay treatment in the prevention of atherogenesis leading to CAD. However, there has been growing research interest in identifying atheroprotective properties of vitamins as a possible supplementation to the established cholesterol lowering treatment regime. Tocotrienol–tocopherol mixed fraction (TTMF) is a member of the vitamin E family. Both tocopherol (TCP) and tocotrienol (T3) have four different isomers (α, β, γ and d) with varying levels of biological activity. Each of these isomers can be differentiated by the number of methyl groups on their aromatic rings (4). Natural sources of TCP are commonly available in vegetable oils such as canola oils, while T3 are found in cereal grains such as oat and barley; rice bran, palm and annatto (90% delta and 10% gamma) oils were described to have some of the richest sources of T3 by Tan and his co-workers (5). TCP differs from T3 by possessing saturated phytyl side chains, while T3 have unsaturated isoprenoid chains along their tails, allowing T3 to have a more potent antioxidant activity as the unsaturated side chains allow them to penetrate tissues with saturated fatty layer more efficiently (6). The side chain of T3 has been shown to alter its membrane distribution and improve interaction with free radicals, compared to TCP. T3 have been demonstrated to have lipid-lowering properties and the potential to reduce non-lipid-related risk factors for cardiovascular diseases. A study by Qureshi et al. had shown that T3 suppressed the HMGCoA reductase activity in normolipaemic and hyperlipaemic chickens (7) and swines (8). Several in vitro studies have reported the potent antioxidative effects of palm oil-derived TTMF (9, 10). Our group had reported that palm oil-derived TTMF reduced oxidative stress and inflammatory markers expression in vitro, which are significant mechanisms in the formation of atherosclerosis. We also reported that these effects are optimal at low-to-moderate concentrations in in vitro studies (11). Furthermore, animal studies have shown that palm-derived TTMF treatment at dose (15 mg/kg) reduced the expression of IL-6 in the aorta of rabbits with established atherosclerosis (12).

Although most studies have strongly supported TTMF as an effective therapeutic agent in the prevention of CAD, not all studies have found this association. Large-scale, double-blind intervention studies failed to demonstrate consistent efficacy of vitamin E against atherosclerosis (13). This is possibly due to the TCP present in vitamin E used in this study, which have been shown to attenuate the anti-atherogenic properties of T3 (14). TCP was reported in several studies to have non-beneficial effects in patients with high risk of developing CAD. TCP protects low-density lipoprotein cholesterol (LDL-c) and tissue from oxidative stress since it is implicated in the development of atherosclerosis and tissues (15). Another group showed that TTMF can preserve the elasticity of internal elastic lamina and cause less intimal thickening (16). This discrepancy has raised concerns about current formulations of vitamin E composition that would be most beneficial in the prevention of atherosclerosis. The possible explanations for these discordant findings could be due to the difference in atheroprotective potency of different T3: TCP proportion. In addition, there is very little understanding in the mechanisms of atheroprotective activities of these various isomers. Furthermore, the effects of T3 on plaque stability in both early and established atherosclerosis have not been well established. Therefore, this study aims to investigate the effects of pure tocotrienol (T3) isomers on atherogenesis with regard to lipid profile as well as looking at their effects on endothelial activation and atherosclerotic plaque stability following initiation of high cholesterol diet (HCD) induced established atherosclerosis and pure T3 supplementation.

## Methods

### Animal and diets

Institutional Animal Ethics approval was obtained prior to initiation of the experiment [Reference Number: 100-FPR(PT 35/9) – ACUC-1/11], and this study was conducted in accordance with guidelines set by the Laboratory Animal Care Unit, UiTM. Thirty New Zealand white rabbits weighing 2.0–2.5 kg (Chenur Sdn Bhd, Malaysia) were randomly divided into early (B1) and established (B2) atherosclerotic group and further subdivided into three intervention arms: T3 4 mg/kg/D (T3–4), 15 mg/kg/D (T3–15) and vehicle without T3 (T3 negative) as negative control. Early atherosclerosis group rabbits were initially fed with 1% HCD for 2 weeks, and established atherosclerosis group rabbits were initially fed with 1% HCD for 8 weeks before randomizing into the three intervention arms and converting to normal diet (ND) with T3 supplementation or without T3(T3 negative) for another 8 weeks. All the animals were fed 100 g for both ND and HCD diet per day and kept under circadian rhythm of 12 h light: 12 h dark with free access to drinking water. Standardization of the living conditions and dietary regime was done to eliminate, as best as possible, confounding factors during the experimentation, and the addition of the T3-negative groups in each intervention arm would further validate that any significant changes observed between intervention groups and their respective T3-negative groups during the intervention were due to the effects of T3.

This study design was adopted from previous published animal studies using the same model (17–20). The reason for giving 1% HCD was to invoke atherogenesis leading up to the formation of either 1) early atherosclerosis defined by microscopic appearance of foam cells without any obvious macroscopic plaque formation and 2) established or advanced atherosclerosis defined by macroscopic appearance of atheromatous plaque. A preliminary experiment conducted by our research team has established that diet enriched with 1% cholesterol given for 2 weeks led to the development of early atherosclerosis, whereas giving 1% HCD for 2 months leads to established/advanced atherosclerosis. The justification for switching both arms to ND following 2 weeks or 2 months HCD is to ensure rabbits survive by the completion of the experimentation period. Previous studies have shown that there were high mortality rates in animals supplemented for long periods with 1–2% cholesterol in their diets due to high hepatotoxicity, which does not allow the animal to survive. Furthermore, massive inflammation in the body does not reflect human pathophysiology (21, 22). Therefore, the switch to ND aimed to sustain the animals long enough to complete the experimentation without hepatotoxicity leading to death.

The standard laboratory ND contained crude protein 18.0%, crude fat 4.0%, carbohydrates 46%, vitamins (carotene, vitamin K, thiamine, riboflavin, niacin, pantothenate, choline, folic acid, pyridoxine, biotin, vitamin B, vitamin A, vitamin D, vitamin E) and minerals (ash, calcium, phosphorous, potassium, magnesium, sulphur, sodium, chlorine, fluorine, ferum, zinc, manganese, copper, cobalt, iodine, chromium, selenium). Both ND and HCD were identical in their chemical composition except that 1% cholesterol was added in the HCD.

### Pure T3 supplement

Pure T3 obtained from annatto plant was supplied by American River Nutrition Inc. It consists of 90% δ-tocotrienol and 10% γ-tocotrienol. All experiments were conducted using pure T3 obtained from the same batch.

### Fasting serum lipid analysis

Fasting blood samples were obtained at baseline and serially at different diet and intervention time points. Blood samples were collected from the marginal ear vein into plain tubes, and serum was separated by centrifugation at 4,000 rpm for 10 min. Serum total cholesterol (TC), high-density lipoprotein cholesterol (HDL-c) and triglycerides (TG) were measured by enzymatic reference methods on an automated analyzer (Cobas 400 PLUS, Roche, USA). LDL-c was analysed by direct method using the same analyzer. Quality control (QC) for all analytes was within recommended reference range prior to analyses.

### Morphological study

Rabbits were euthanized with sodium pentobarbital at the end of the study. The aortas were obtained by dissecting the entire length of the aorta from the ascending aorta up to the bifurcation of the iliac arteries. Longitudinal incision was done on the respective aortas and pinned with the endothelial surface facing up against a polystyrene board. The endothelial surface was then stained with Sudan IV and photographed. Three different sites of aortic tissue were sampled as follows: 10 mm away from the aortic arch, 10 mm away from upper thoracic and 10 mm above iliac bifurcation, with each section measuring 15 mm in length. The percentage of Sudan IV-stained atherosclerotic regions were analysed quantitatively using image analysis software (analySIS^®^ FIVE, Olympus, USA). The percentage of atherosclerotic lesions were calculated as the percent of the lesion area to total aortic area.

### Immunohistochemistry staining

Rabbits’ aortic specimens were fixed in 10% formalin solution and underwent automated tissue processing procedure over night. The processed tissues were embedded in paraffin and sectioned into 3-µm thick tissue sections. The tissue sections were placed on coated slides, deparaffinised and hydrated gradually. All slides were immersed in 3% hydrogen peroxide in methanol for 15 min at room temperature to block endogenous peroxidase activity. The slides were then rinsed and subjected to microwave for antigen retrieval. After 20 min of pre-incubation with blocking serum at room temperature, the sections were further incubated with rabbit polyclonal antibody against E-selectin, VCAM-1, ICAM-1, IL-6, MMP-9 and MMP-12 (Santa Cruz Biotechnology, CA, USA; R 1:100 TO 1:250 dilution range) at room temperature for 1 h. Sections were then incubated with biotinylated secondary antibody (Santa Cruz Biotechnology, CA, USA) followed by peroxidase-conjugated streptavidin for 30 min at room temperature each. The peroxidase was visualized by incubation with diaminobenzidine in the dark for 10 min. Blind evaluations of immunostaining were made by a trained histopathologist. Image quantification was made based on defined brown endothelial staining by using soft imaging solution software (analySIS^®^ FIVE, Olympus, USA). The percentage of positivity staining was determined by endothelial cell stained positive (brown) to total lesion area. For negative controls, incubation steps of primary antibody were omitted.

### Statistical analysis

The sample size of this study (*n*=5) was determined by the Power and Sample size program (PS) version 3.0 to provide a study power of 80% at 5% level of significance. Variables with normal distribution were expressed as mean±SEM. Comparison between three groups was determined by Kruskall–Wallis H test. Paired sample *t*-test was used to determine the effects of treatment in each treatment group. For comparison between more than two treatment groups, non-parametric test Wilcoxon–Mann–Whitney was used. The criterion for statistical significance was at *p<*0.05. Data were analysed by a statistical package program (SPSS) version 17.0.

## Results

### Fasting serum lipid

Tables 1 and 2 summarize the fasting serum lipids of the rabbits in the early and established atherosclerosis experimental arms, respectively. There was significant %Δ reduction in both T3–4 mg/kg and T3–15 mg/kg groups compared to T3-negative rabbits group in the established atherosclerosis experimental model (mean±SEM: −9.8±15.7 and −36.7±11.8% vs. −61.1±1.6%, *p<*0.05 and *<*0.01, respectively). %Δ reduction was also observed with serum TC concentration in the T3–4 mg/kg group compared to T3-negative rabbits group (mean±SEM: −44.2±11.1 vs. −65.3±0.8%) in the established atherosclerosis experimental arm. However, there were no significant differences observed in TG and HDL-c between both T3 intervention groups and T3-negative arm in established atherosclerosis. There were no differences observed between the groups in terms of lipid profile in the early atherosclerosis experimental arm.

**Table 1:** Fasting serum lipid concentrations in B1

	B0			W2 post-HCD			W6 post-ND +T3			W10 post-ND+T3		
				
B1 Group	T3-negative rabbits group (*n*=5)	T3–4 mg/kg (*n*=5)	T3–15 mg/kg (*n*=5)	T3-negative rabbits group (*n*=5)	T3–4 mg/kg (*n*=5)	T3–15 mg/kg (*n*=5)	T3-negative rabbits group (*n*=5)	T3–4 mg/kg (*n*=5)	T3–15 mg/kg (*n*=5)	T3-negative rabbits group (*n*=5)	T3–4 mg/kg (*n*=5)	T3–15 mg/kg (*n*=5)
Absolute level (mean±SEM)												
TC	0.8±0.1	1.7±0.2	1.3±0.1	19.9±1.8^a^	35.4±4.4^a^	30.0±1.7^a^	2.9±1.5^b^	1.0±0.3^b^	1.8±0.3^b^	0.6±0.1^b^	0.8±0.0^a^,^b^	0.68±0.1^a^,^b^,^c^
LDL-c	0.3±0.0	0.8±0.3	0.7±0.0	22.2±4.2^a^	27.1±6.9^a^	28.3±1.7^a^	2.4±1.5^b^	0.4±0.2^b^	1.2±0.3^b^	0.1±0.0^a^,^b^	0.2±0.0^b^	0.2±0.0
TG	0.6±0.2	0.6±0.0	0.7±0.1	1.1±0.3	1.3±0.2^a^	0.9±0.1^a^	0.9±0.2	1.0±0.2	0.6±0.0^b^	0.6±0.0	0.7±0.1	0.6±0.1^b^
HDL-c	0.4±0.0s	0.6±0.1	0.7±0.1	4.0±0.7^a^	4.0±1.3^a^	5.0±0.3^a^	0.7±0.2^b^	0.3±0.1^b^	0.7±0.0^b^	0.3±0.1^b^	0.6±0.0^b^,^c^	0.4±0.1^b^,^c^
%Δ reduction (mean±SEM)												
TC							−85.0±7.6	−97.2±s1.1	−94.0±1.0	−13.0±6.8	−16.6±16.1	−30.7±4.5
LDL-c							−97.0±0.5	−97.5±0.2	−97.7±0.3	−39.4±12.7	−36.7±14.6	−33.6±8.3
TG							−90.8±5.1	−98.5±0.8	−95.7±1.1	−83.9±3.9	−90.1±3.6	−85.5±0.8
HDL-c							−99.6±0.1	−99.0±0.4	−99.4±0.2	−92.1±1.8	−80.5±4.4	−91.8±1.6

TC: total cholesterol; LDL-c: low-density lipoprotein cholesterol; TG: triglycerides; HDL-c: high-density lipoprotein cholesterol; HCD: high cholesterol diet.

Absolute concentrations and percent change of TC, HDL-c, LDL-c and TG in T3-negative rabbits group and T3–4 mg/kg and T3–15 mg/kg groups. For absolute data, concentration

a indicates significant differences compared to baseline

b compared to week 2 and

c compared to week 6. Results are expressed as mean±SEM.

**Table 2:** Fasting serum lipid concentrations in B2

	B0			W4 post-HCD			W8 post-HCD			W12 post-ND+T3			W16 post-ND+T3		
					
B2 Group	T3-negative rabbits group (*n*=5)	T3–4 mg/kg (*n*=5)	T3–15 mg/kg (*n*=5)	T3-negative rabbits group (*n*=5)	T3–4 mg/kg (*n*=5)	T3–15 mg/kg (*n*=5)	T3-negative rabbits group (*n*=5)	T3–4 mg/kg (*n*=5)	T3–15 mg/kg (*n*=5)	T3-negative rabbits group (*n*=5)	T3–4 mg/kg (*n*=5)	T3–15 mg/kg (*n*=5)	T3-negative rabbits group (*n*=5)	T3–4 mg/kg (*n*=5)	T3–15 mg/kg (*n*=5)
Absolute level (mean±SEM)															
TC	1.2±0.1	1.2±0.1	1.2±0.1	80.4±5.1^a^	50.2±7.9	76.6±3.3^a^	47.3±0.6^a^,^b^	69.8±3.1	60.0±2.2^a^,^b^	16.4±0.3^a^,^b^,^c^	42.0±5.8	23.6±1.7^a^,^b^,^c^	7.1±0.7^a^,^b^,^c^,^d^	12.5±2.9	7.8±0.8^a^,^b^,^c^,^d^
LDL-c	0.2±0.0	0.3±0.1	0.2±0.1	74.8±12.5^a^	49.4±10.1	87.5±10.0^a^	41.7±1.8^a^	44.5±5.3	41.6±5.8^a^,^b^	16.1±0.3^a^,^b^,^c^	42.0±1.8	23.8±1.3^a^,^b^	7.4±0.3^a^,^b^,^c^,^d^	13.3±4.1	7.4±0.8^a^,^b^,^c^,^d^
TG	0.5±0.0	0.6±0.1	0.5±0.0	2.1±0.3^a^	1.5±0.2	2.3±0.2^a^	1.8±0.5^a^	1.8±0.1	2.1±0.3^a^	0.8±0.2^b^,^c^	1.0±0.1	1.0±0.0^a^,^b^,^c^	0.7±0.2^b^,^c^	0.7±0.1	0.6±0.1^b^,^c^,^d^
HDL-c	0.9±0.1	1.0±0.0	0.9±0.1	7.3±0.2^a^	6.6±0.2	7.1±0.4^a^	5.3±0.3^a^	6.1±0.7	5.0±0.2^a^,^b^	1.6±0.0^a^,^b^,^c^	2.6±0.3	2.1±0.3^a^,^b^,^c^	1.0±0.0^b^,^c^,^d^	2.0±0.4	0.9±0.1^a^,^b^,^c^,^d^
Percent change (mean±SEM)															
TC										−65.3±0.8	−44.2±11.1*	−60.1±4.1	−85.0±1.6	−82.5±3.7	−87.0±1.7
LDL-c										−61.1±1.6	−9.8±15.7*	−36.7±11.8**	−82.1±1.2	−65.5±14.2	−81.2±2.2
TG										−50.6±10.6	−41.8±7.4	−45.7±9.4	−55.8±6.7	−61.3±8.6	−71.3±4.9
HDL-c										−69.3±1.8	−55.1±7.0	−57.3±7.2	−80.2±0.8	−66.1±7.7	−81.1±3.0

TC: total cholesterol; LDL-c: low-density lipoprotein cholesterol; TG: triglycerides; HDL-c: high-density lipoprotein cholesterol; HCD: high cholesterol diet.

Absolute concentration and percent change of TC, HDL-c, LDL-c and TG in T3-negative rabbits group and T3–4 mg/kg and T3–15 mg/kg groups. For absolute data, concentration

a indicates significant differences compared to baseline

b compared to week 4

c compared to week 8 and

d compared to week 12. For percent change

* indicates p < 0.05 and

** indicates p < 0.01 significant differences compared to T3 negative rabbits group of each week. Results are expressed as mean±SEM.

### Measurement of atherosclerotic lesion

**Fig. 1:**
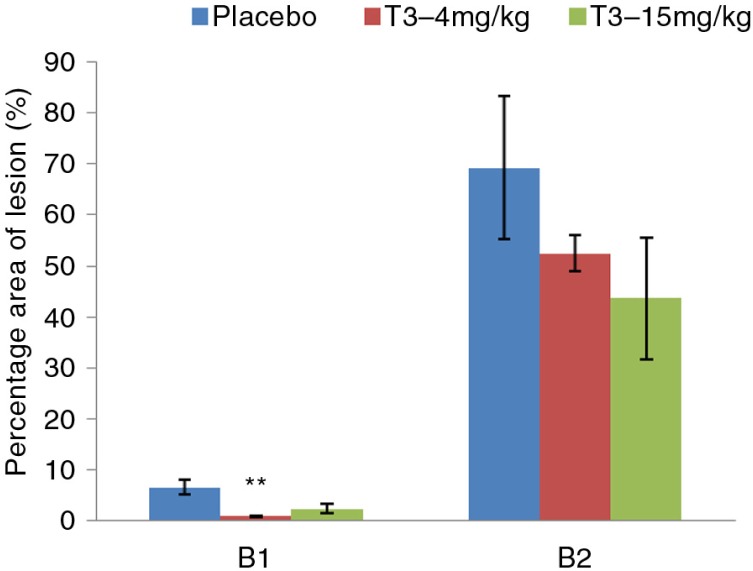
Quantitative analysis of atherosclerotic lesion in early and established atherosclerotic rabbit groups. The lesion area (Sudanophilic area) in each aortic segment was determined using soft imaging solution software (analySIS^®^ FIVE, Olympus, USA). Data are expressed as mean±SEM. **p<0.01 compared to T3-negative rabbits group.

Atherosclerotic lesions in B1 showed significant reduction in T3–4 mg/kg group as compared to T3-negative rabbits group (mean±SEM: 0.9±0.1% vs. 6.6±1.4% *p*=0.008). T3–15 mg/kg group showed a reduction trend but did not reach statistical significance when compared to T3-negative rabbits group (mean±SEM: 2.4±0.9% vs. 6.6±1.4% *p*=0.1). B2 showed a trend in atherosclerotic lesion area reduction in both 4 mg/kg and T3–15 mg/kg treatment groups but did not reach statistical significance (mean±SEM: 525±3.6% vs. 69.2±14.0% *p*=1.0 and 43.6±12.0% vs. 69.2±14.0% *p*=1.15 respectively). (Fig. 1).

### Tissue biomarkers expression

**Fig. 2:**
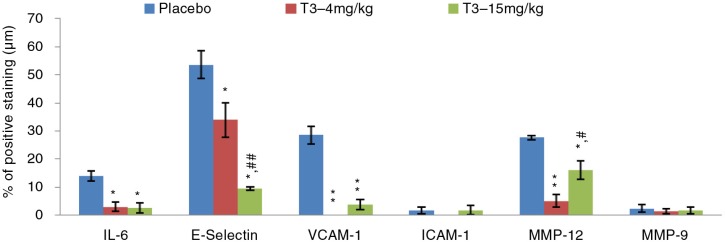
IHC expression of biomarkers in pure T3–4 mg/kg and T3–15 mg/kg treatment groups versus T3-negative rabbits group in early atherosclerosis. Data expressed as mean±SEM. (*) and (**) indicate significant differences (*p<*0.05 and *p<*0.01, respectively) between T3 and T3-negative rabbits group. (#) and (##) indicate significant differences (*p<*0.05 and *p<*0.01, respectively) between T3–4 mg/kg and T3–15 mg/kg.

There was a reduction in aortic tissue expression of VCAM-1 (0.2±0.0% vs. 28.5±3.14%; **p=0.008), IL-6 (3.0±1.7% vs. 14.0±1.7%; *p=0.032) and MMP-12 (5.2±2.2% vs. 27.7±0.8%; **p=0.008) of T3–4 mg/kg treatment group compared to T3-negative rabbits group in the B1 experimental arm (Fig. 2). In addition, T3–15 mg/kg showed significant reduction in VCAM-1 (3.9±1.9% vs. 28.5±3.1%; **p=0.008), E-selectin (9.5±0.6% vs. 53.6±5.0%; **p=0.008), IL-6 (2.6±1.7% vs. 14.0±1.7%; *p=0.016) and MMP-12 (16.2±3.2% vs. 27.7±0.8%; *p=0.016) compared to T3-negative rabbits group in the same experimental arm.

**Fig. 3:**
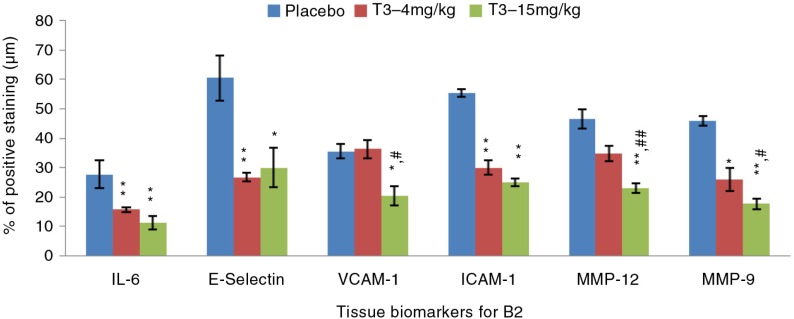
IHC expression of biomarkers in pure T3–4 mg/kg and T3–15 mg/kg treatment groups versus T3-negative rabbits group in established atherosclerosis. Data are expressed as mean±SEM. (*) and (**) indicate significant differences (*p<*0.05 and *p<*0.01, respectively) between T3 and T3-negative rabbits group. (^#^) and (^##^) indicate significant differences (*p<*0.05 and *p<*0.01, respectively) between T3–4 mg/kg and T3–15 mg/kg.

Almost similar findings were observed in the established atherosclerosis group (B2), where T3–4 mg/kg showed a reduction in aortic tissue expression of ICAM-1 (29.9±2.4% vs. 55.3±1.3%; **p=0.008), E-selectin (26.7±1.5% vs. 60.5±7.6%; **p=0.008), IL-6 (15.7±0.7% vs. 27.7±4.8%; **p=0.008), MMP-12 (34.8±2.7% vs. 46.5±3.4%; **p=0.008) and MMP-9 (25.9±3.9% vs. 45.9±1.7%; **p=0.008) compared to T3-negative rabbits group (Fig. 3). T3–15 mg/kg treatment group also demonstrated reduced VCAM-1 (20.5±3.3% vs. 35.6±2.5%; *p=0.016), ICAM-1 (24.9±1.3% vs. 55.3±1.3%; **p=0.008), E-selectin (29.9±6.7% vs. 60.5±7.6%;*p=0.032), IL-6 (11.3±2.2% vs. 27.7±4.8%; **p=0.008), MMP-12 (23.0±1.7% vs. 46.5±3.4%; **p=0.008) and MMP-9 (17.6±1.9% vs. 45.9±1.7%; **p=0.008) compared to T3-negative rabbits group.

## Discussion

The objective of this study was to investigate the cardioprotective effects of pure T3 (δ-90%, γ-10%) in the treatment of early (B1) and established (B2) atherosclerosis in terms of its lipid-lowering properties and its effects on tissue expression of markers of inflammation, endothelial activation, oxidative stress and plaque stability. In order to induce early and established atherosclerosis, 1% HCD was given for 2 and 8 weeks, respectively, as has been used in previous rabbit experimental models to generate atherosclerosis (17, 18). The present data of B1 and B2 showed expected increment in lipid parameters following HCD for 2 and 8 weeks, respectively, which were similarly noted in previous animal studies (17). All groups were then treated with either 4 or 15 mg/kg T3 and switched to ND for another 8 weeks. Analyses of lipid profile and atherosclerotic lesions were performed, while expressions of endothelial activation, inflammation and plaque stability were evaluated using immunohistochemistry analysis.

This present study demonstrated lipid-lowering properties of pure T3 in the established atherosclerosis group with percent change reductions of TC in the T3–4 mg/kg dose treatment group and LDL-c in both T3–4 mg/kg and T3–15 mg/kg dose treatment groups. Our findings concurred with a previous report by Zaiden et al., which demonstrated reduction in serum TC and TG concentrations following 4 weeks treatment with 50 mg/kg pure gamma-delta T3 (18). Qureshi et al. also reported similar lipid-lowering properties of T3 in corn soy-fed chicken treated with δ-T3 for 4 weeks that significantly reduced TC and LDL-c (21). However, lipid-lowering properties were not seen in the early atherosclerosis experimental arm given 2 weeks of HCD. This could be partly attributed to the short duration of HCD feeding that may not have elevated lipid concentrations high enough to demonstrate significant lipid-lowering following treatment with T3. Another factor that could explain the absence of significant differences seen in lipid profile was the relatively short experimental period that may not have captured the full lipid-lowering effects in this experimental arm.

Despite the neutral effects in lipid lowering observed in the early atherosclerosis arm, significant plaque regression was noted in the 4 mg/kg T3 treatment group of this arm (mean±SEM: 0.9±0.1% vs. 6.6±1.4%; *p*=0.008), which was not observed in the 15 mg/kg T3 treatment arm. There is still a knowledge gap with regard to the rationalization for the regression of atherosclerotic plaque seen with lower dose T3. However, this finding is consistent with a previous in vitro study done by Nawawi et al., highlighting γ- and δ-T3 optimally reduced soluble protein expression of IL-6 and ICAM-1 at lower concentration (23). Plaque regression was reported by one study using vitamin E (T3: TCP ratio not clearly stated) in the treatment of HCD-induced rabbits, which reported significant reduction in atherosclerotic lesion (24). Another study by Nafeeza et al. used TTMF with a ratio of T3:TCP (70:30) throughout the experiment and showed atherosclerotic plaque regression following 10 weeks of 2% HCD-induced atherosclerosis rabbit model (16). However, Muhammad et al. reported conflicting results exhibiting no significant reduction in atherosclerotic plaques of TTMF treated 1% HCD-induced early and established atherosclerosis rabbit groups (17). These discrepancies are probably due to the different experimental designs, percentage HCD used and ratio of T3:TCP, whereby the higher α-TCP content in the mixture could possibly interfere with the beneficial effects of pure T3.

Endothelial activation is the initial event of atherosclerosis development and is activated by expression of multiple cell adhesion molecules including VCAM-1, ICAM-1 and E-selectin. This study demonstrated reduction in tissue expression of adhesion molecules in both treatment groups of the B1 and B2 arms compared to T3-negative rabbits group. These findings are consistent with previous cell culture reports on the favourable effects of T3 as a treatment measure in reducing expression of adhesion molecules (24–28). An earlier study by Theriault et al. reported tumor necrosis factor (TNF)-α-stimulated human endothelial cells treated with α-T3 at a dose of 25 µmol/l significantly decreased ICAM-1 and E-selectin expressions (25). A comparison study by Noguchi et al. found that α-T3 exhibited a 10-fold higher reduction in VCAM-1 expression than α-TCP in human endothelial cells pre-treated with TNF-α (26). Naito's group reported significant inhibition of VCAM-1 mRNA expression and reduction of cell monocytic adhesion following 18 h administration of α-, β-, γ- and δ-T3 in 25-hydroxycholesterol induced human coronary artery endothelial cells (HCAEC) (28). These findings suggest that reductions in monocytic cell adhesion are associated with the decrease in adhesion molecules expression with δ-T3 showing the most reduction in monocytic cell adhesion compared to α-, β- and γ-T3. This was further supported by Chao et al. who reported that δ-T3 is the most effective T3 isomer followed by γ-T3 in reducing VCAM-1 and E-selectin expressions. An in vivo study by Koga et al. also reported similar findings with vitamin E showing a reducing trend in ICAM-1 and VCAM-1 tissue expressions in HCD-induced atherosclerotic rabbits.

Interleukin-6 (IL-6) is one of the main cytokines produced by activated macrophages during inflammation. The over-expression of IL-6 is seen in many atherosclerosis studies (29, 30). Suppression of IL-6 expression is believed to play an important role in preventing further development of atherosclerosis. This study demonstrated reduction in IL-6 tissue expression in all T3 treatment groups in both B1 and B2 arms compared to T3-negative rabbits group. These findings are consistent with previous reports of favourable anti-inflammatory effects of T3 (31–33). Ng and Ko reported TTMF comprised of 50% T3, 15% TCP and 25% other compounds, significantly reduced IL-6 expression in lipopolysacharide (LPS)-stimulated cells (31). Another study by Yam et al. showed δ-T3 to be the most potent isomer in reducing IL-6 expression in LPS-stimulated cells (32). LPS is widely used in cell culture studies to induce inflammatory response (34). These findings are in parallel with Qureshi's group where low concentrations of δ-T3 blocked LPS-gene-induced gene expression of IL-6 (33).

Proteolytic enzyme MMPs, produced by macrophage-derived foam cells stimulated by inflammatory process, play an important role in plaque destabilization (35). Increasing evidence shows that MMPs are over-expressed in atherosclerotic lesions as a consequence of inflammation. There is therapeutic interest targeting the inhibition of MMP-9 and MMP-12 expressions, which have been shown to degrade extracellular matrix and reduce fibrous cap of atheromatous plaques (36–39). Both T3 treatment groups in B1 showed significant reduction in MMP-12 with the 4 mg/kg arm having the most reduction. This could explain the significant atherosclerotic plaque regression observed in this arm as decreased MMP-12 leads to reduced fibrous cap degradation forming a more stable plaque, which is defined by higher fibrous cap to lipid core ratio. The lesser lipid core will cause decreased Sudan-V staining of these plaques in this group.

However, there was no significant difference in MMP-9 between the two groups which could be attributable to the fact that MMP-9 was not over-expressed in early atherosclerosis and therefore any reduction seen did not reach statistical significance. This is in keeping with another study by Das et al. which showed δ-T3 treatment group showed no effects in reducing MMP-9 mRNA expression in HCD-induced rabbits (40). Similarly, a study done by Muhammad et al. showed no difference in MMP-9 between the TTMF (T3: TCP, 70%; 30%) treatment group and T3-negative rabbits group in HCD-induced early atherosclerosis rabbits (17). These consistent findings suggest that in early atherosclerosis, changes in MMP-9 following treatment with T3 may not be significant enough as they are not overly expressed following HCD. However, both T3 treatment groups in established atherosclerosis significantly reduced MMP-9 and MMP-12 tissue expression compared to T3-negative rabbits group suggesting its potential role in attenuating progression of plaque instability.

Due to technical and financial constraints, we were unable to further explore correlation between plasma T3 with endpoint analyses, which would further validate that the atheroprotective effects observed were due to T3. Future studies determining this would close the knowledge gap regarding the bioavailability of T3, particularly the varying T3 isomers.

## Conclusion

The findings of this study highlight the lipid-lowering potential of pure T3 in HCD-induced atherosclerotic rabbit model, particularly in the established atherosclerosis arm. Furthermore, T3 appears to reduce atherosclerotic lesion, and tissue expression of biomarkers of endothelial activation and inflammation, which are key processes of atherogenesis. In addition, T3 appears to increase plaque stability through the reduction of proteolytic enzymes such as MMP-9 and MMP-12. These suggest its potential role as an atheroprotective agent in early and established atherosclerosis. The findings of this study further demonstrate that atheroprotective effects are more prominently observed in established than early atherosclerosis and that the higher dose of 15 mg/kg body weight showed higher potency. Further studies determining T3's bioavailability, correlation between plasma T3 isomers with endpoint analyses and extending this research over a longer treatment duration with progression to clinical trial would further confirm the cardioprotective benefits of T3 and whether or not it is potentially useful as an adjunct therapy to standard treatment regimes in the prevention of atherosclerosis and its related complications.
